# Laryngeal Phthisis, or Consumption of the Throat

**Published:** 1901-05

**Authors:** 


Laryngeal Phthisis, or Consumption of the Throat. By Richard Lake, F.R.C.S., Surgeon to Metropolitan Throat Hospital, London. Published by P. Blakiston's Son & Co., Philadelphia. Price, $2.00.

This is a small brochure of ninety pages, neatly arranged in an attractive binding, and containing some most excellent colored plates of the various forms of phthisis of the larynx. The first sentence of the author's introduction clearly sets forth the purpose of its publication. "The importance of this subject and the immense value of the knowledge of its treatment, induced me to prepare this small work, less as a treatise than as the record of results obtained by the observation and treatment of over 300 cases." Mr. Lake as Surgeon Laryngologist to North London Hospital for Consumption has had unusual advantages of treating a large number of cases and of putting into practice any new method of treatment promulgated, and thus deciding its value or its worthlessness. The subject is quite exhaustively treated in every respect, and coupled with a tabulated report of 300 cases this small work is made exceedingly readable and of value to both physician and laryngologist. The colored plates are very good, and while not perhaps equaling those found in some of the larger text-books, they are yet more numerous as illustrative of the subject in question. Laryngeal phthisis is always an interesting subject, and as such frequently tries the ability of the best laryngologist. Any helps added to a better understanding of the subject should always be well received.

